# Supplementary material for: Slow RNAPII Transcription Elongation Rate, Low Levels of RNAPII Pausing, and Elevated Histone H1 Content at Promoters Associate with Higher m6A Deposition on Nascent mRNAs
Source: Genes (Basel). 2022 Sep 14;13(9):1652. doi: 10.3390/genes13091652 (PMC9498810; doi:10.3390/genes13091652)
Supplement: Supplementary file 1 [file genes-13-01652-s001.zip › Table S1_Gallego et al.pdf]

Supplementary Table S1. Main distribution data (mean, median, SD) of the variables analyzed across slow, medium and fast rate genes.

(A) Elongation rates of slow, medium and fast rate categories (**Figure 1D**).

Distribution statistics	mean	median	SD
**WT slow**	1.741578	1.82	0.6204964
**WT medium**	3.047892	3.06	0.1980922
**WT fast**	4.149014	3.7	1.098192
**H1-TKO slow**	1.621007	1.58	0.5870089
**H1-TKO medium**	3.189032	3.24	0.270798
**H1-TKO fast**	4.276415	3.96	0.931213

(B) RNAPII levels at TSS and transcription elongation rate categories (**Figure 2C**).

Distribution statistics	mean	median	SD
**WT slow**	2.078333	1.796961	1.519135
**WT medium**	2.910465	2.65276	1.573692
**WT fast**	3.096705	2.883417	1.808997
**H1-TKO slow**	2.268346	1.949536	1.835134
**H1-TKO medium**	3.173807	2.916454	1.704815
**H1-TKO fast**	3.373803	3.149902	1.88342

(C) CheRNA levels and transcription elongation rate categories (**Figure 2D**).

Distribution statistics	mean	median	SD
**WT slow**	0.6778481	0.3967252	2.671459
**WT medium**	1.33611	0.9492873	1.560947
**WT fast**	1.64832	1.23254	1.59928
**H1-TKO slow**	0.6964565	0.4051067	2.879507
**H1-TKO medium**	1.254151	0.8773069	1.424471
**H1-TKO fast**	1.593306	1.214152	1.542872

(D) RNAPII pausing and transcription elongation rate categories (**Figure 2E**).

**Distribution statistics**	**mean**	**median**	**SD**
**WT slow**	4.047321	3.601417	2.444012
**WT medium**	4.729108	4.440732	2.10001
**WT fast**	4.494256	4.269058	2.02727
**H1-TKO slow**	3.804525	3.476232	2.12269
**H1-TKO medium**	4.547046	4.330952	1.977218
**H1-TKO fast**	4.429188	4.234174	2.036129

(E) m6A levels on nascent RNAs and transcription elongation rate categories (**Figure 2F**).

**Distribution statistics**	**mean**	**median**	**SD**
**WT slow**	1.171492	0.587628	12.37888
**WT medium**	0.7106489	0.5421378	4.49058
**WT fast**	0.6123346	0.5321565	0.9104364
**H1-TKO slow**	0.8846519	0.5113616	11.65021
**H1-TKO medium**	0.8490443	0.4763778	9.28557
**H1-TKO fast**	0.7145619	0.4918678	6.316083

(F) H1c and H1d levels and transcription elongation rate categories (**Figure 3B**).

**Distribution statistics**	**mean**	**median**	**SD**
**H1c slow**	15.85641	14.51838	18.14094
**H1c medium**	13.59969	13.08446	4.640742
**H1c fast**	13.62549	13.08446	4.34269
**H1d slow**	17.18394	16.14409	19.29534
**H1d medium**	15.49293	15.14754	3.599277
**H1d fast**	15.50367	15.14754	3.510373

(G) METTL3 levels and transcription elongation rate categories (**Figure 3C**).

**Distribution statistics**	**mean**	**median**	**SD**
**TSS slow**	135.5698	125	119.8566
**TSS medium**	139.1475	131	60.1823
**TSS fast**	142.4269	134	56.43799
**TTS slow**	85.46481	74	73.24369
**TTS medium**	84.35304	73	74.21311
**TTS fast**	83.80666	75	44.11249
