# Supplementary figures and images for: Slow RNAPII Transcription Elongation Rate, Low Levels of RNAPII Pausing, and Elevated Histone H1 Content at Promoters Associate with Higher m6A Deposition on Nascent mRNAs
Source: Genes (Basel). 2022 Sep 14;13(9):1652. doi: 10.3390/genes13091652 (PMC9498810; doi:10.3390/genes13091652)

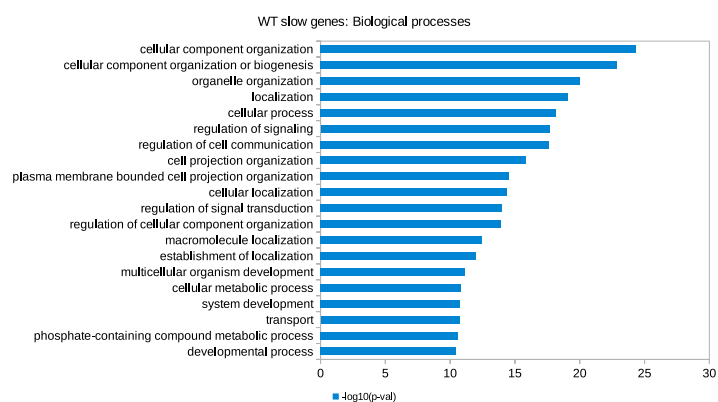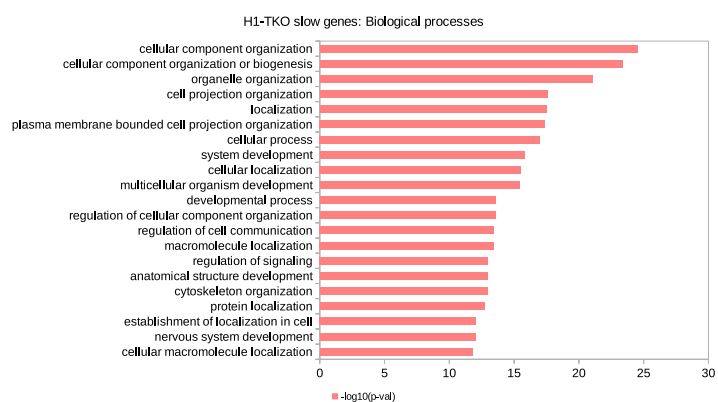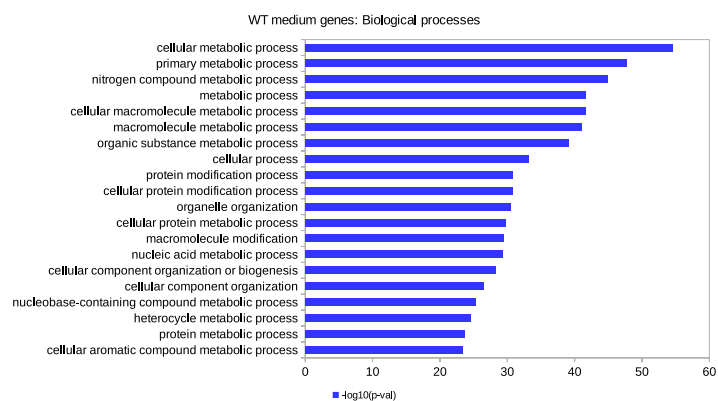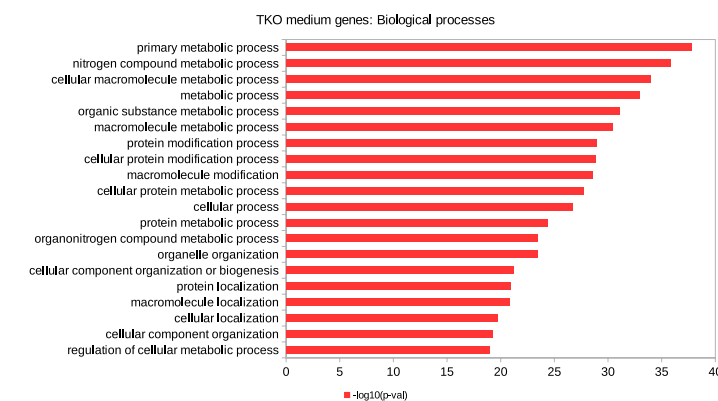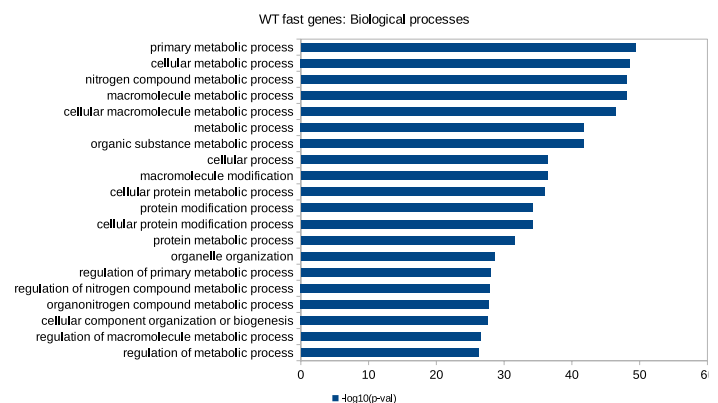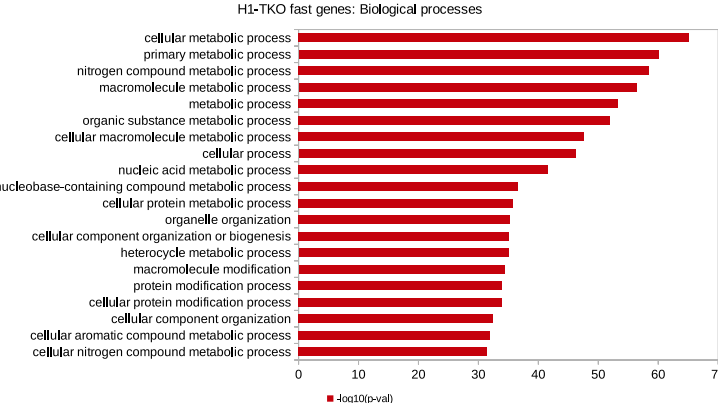

Supplementary Figure S2. Go-terms of the gene-rate groups in WT and H1-TKO mESCs.

Supplement: Supplementary file 1 [file genes-13-01652-s001.zip › Figure S2_Gallego et al.pdf]
